# ‘A British Problem Affecting British People’: Sickle Cell Anaemia, Medical Activism and Race in the National Health Service, 1975–1993

**DOI:** 10.1093/tcbh/hwab007

**Published:** 2021-07-17

**Authors:** Grace Redhead

**Affiliations:** Department of History, University of Warwick, UK

## Abstract

Recent historiography has explored a contradiction at the heart of the British welfare state—it was founded on and supported by migrant and non-white labour, whose own healthcare and broader welfare state entitlements were neglected. This article explores how this contradiction was exposed and challenged by some of the health service’s own workforce, who witnessed and contested racism in the National Health Service (NHS). This is discussed through the lens of the treatment of sickle cell anaemia (SCA), a genetic trait and disease more common in people of African, South Asian, Middle Eastern and Mediterranean descent, which has been highly racialized as affecting black people in particular. By pushing for improved responses to pain in sickle cell disease, and demonstrating the need for SCA screening in urban areas, healthcare professionals within the NHS—many of whom were black or migrant nurses, health visitors or doctors—articulated the status and entitlements of Black British citizenship.

On 5 August 1986, a twenty-seven-year-old black man, Stephen Bogle, was taken ill at a chip shop in Hackney, London. An ambulance was called, which Bogle refused to get into, and soon a police officer arrived and took him to Hackney Police Station. There, the officer learned Bogle was the subject of an outstanding arrest warrant for minor driving charges and possession of ‘a small amount of cannabis’, and he was remanded in custody for a week at Thames Magistrates Court. In the hospital wing at Brixton Prison, medical staff discovered that Bogle had a history of mental illness and also the genetic blood disorder sickle cell disease (SCD). His fluid balance chart from this hospital stay showed a substantial loss of fluid, which was not corrected despite the risk that dehydration poses to SCD patients.^1^ He was pronounced fit to attend court on 12 August, and—unable to stand—was transported in a wheelchair to Thames Magistrates Court where he was placed in a court cell at 11 a.m.^2^ Several witnesses at the court subsequently testified that police officers had called Bogle ‘a malingerer’, not ‘co-operative’ and was in a wheelchair because he was ‘able but not willing to walk’.^3^ A police surgeon called for an ambulance but when they returned, Bogle was not breathing, and he died shortly before 1 pm.^4^ An inquest found that Bogle died from ‘natural causes aggravated by a lack of care’.^5^ In his last days, Stephen Bogle was caught between two branches of the post-war British state—the criminal justice system and the health service. In the Hackney chip shop, he had been claimed by both a police car and an ambulance when he was first taken ill. In the custody of the carceral state, his physical illness was interpreted as resistance to police intervention and reluctance to face justice, and he died as a result.

This article argues that the choice Bogle faced between the ambulance and the police car was not as stark as it might appear—that Bogle died not because he was being tended to by police, but because both branches of the state would read him and his health condition as non-compliant and undeserving rather than seriously unwell. It will explore how sickle cell anaemia (SCA) was seen by the NHS, both at the level of local commissioning and in encounters in medical spaces.^6^ In his study of citizenship in post-war Britain, Matthew Grant notes that ‘although non-white people may have had the status of formal citizens, they were not treated as such, politically or socially’.^7^ In practice, the post-war British welfare state, and the NHS in particular, made black communities in Britain legible as criminal or social issues or irrelevant, expensive minorities, obscuring their public health needs and invalidating them as patients.^8^ As health policy transitioned from universalist public health initiatives to a clinical focus on individual idiosyncrasy, ethnic minorities qualified for neither large-scale public health campaigns, nor for personalized clinical attention.^9^ A case study of SCA illustrates the contradictory and intertwined politics of the welfare state and race in postcolonial Britain, which has received increasing historiographical attention. This article engages with two strands of this literature at a crucial point of their intersection. 

The first of these strands has explored how non-white people and migrants have been tacitly excluded from notions of entitlement to welfare state assistance. Robbie Shilliam has shown that a longstanding racialized division between the ‘deserving’ and the ‘undeserving’ poor in Britain endured in the post-war welfare state, in an informal ‘colour bar’ separating the so-called white working class from immigrant Commonwealth workers.^10^ As Camilla Schofield has shown in her study of late 1960s and early 1970s Powellism, the welfare state was understood as a reward for a wartime sacrifice which was, in turn, ‘whitewashed’ by Enoch Powell and his supporters, erasing ‘the sacrifices of Britain’s non-white colonial forces’ and refusing their welfare entitlement.^11^ This manifested in a jealously guarded welfare system, with immigration law and the welfare state often used to delimit one another. The progressive legal exclusion of Commonwealth migrants in the 1960s and 1970s, marked by increasingly restrictive Commonwealth Immigration Acts, was partially predicated on and fuelled by discourses of ‘welfare parasitism’, a fear that such migrants would exploit and overrun the welfare state.^12^ As Paul Gilroy has observed, when British political regimes were ‘in flight from socialist principles and welfare state inclusivity’ in the late 1970s and 1980s, they used notions of ‘strangers and aliens’ to measure the limit for welfare.^13^ From the first influx of Commonwealth migrants, Roberta Bivins has argued, migrant health was often a subject of intense scrutiny, seen as both a research opportunity and an existential threat for the British health service.^14^ Jordanna Bailkin has argued that migration ‘was one of the first issues that postwar experts were called upon to manage and define’, and that their focus on migrants fundamentally shaped British social services.^15^

The second strand of this historiography has shown that empire, decolonization and migration made the welfare state possible and shaped its forms of assistance in myriad ways. The NHS was explicitly framed by William Beveridge as a mission that might guarantee the legacy of great ancestors such as the slave-trader Francis Drake, and ensure the health of ‘the best of our breed’.^16^ Once the NHS was established, Britain depended upon its former colonial territories to ‘provide the means of furnishing welfare benefits to its metropolitan citizens’, as Nadja Durbach has illustrated in her study of welfare state orange juice, derived from oranges grown in the Caribbean.^17^ Caribbean and Asian migrants have played a significant role in staffing the NHS since it first opened its doors. Despite increasingly strict immigration controls, migrant doctors and nurses were still seen as essential—they could be employed at cheaper rates and used to cushion white British doctors from the ‘rottenest, worst’ positions in the sector.^18^ In 1968, Commonwealth migrants made up 30 per cent of nurse pupil vacancies and 29 per cent of student midwives; by 1975 18 per cent of GPs and 32 per cent of those working in the hospital service were born outside Britain, and 50 per cent of migrant doctors were born in the Indian subcontinent.^19^ These medical workers played a significant role in shaping health services in the NHS, particularly primary care, geriatrics and mental health services.^20^ This labour meant that the NHS became ‘a site in which racial and ethnic inclusion and British diversity could be recognised and portrayed’.^21^

Taken together, these two strands of analysis illustrate a long-standing contradiction at the heart of the British welfare state—founded on and supported by migrant and non-white labour, but who were excluded from the imagined community of public health. This article explores how this contradiction was identified and tackled by some of the health services’ own workforce. Kennetta Hammond Perry argues that Caribbean migrants to Britain made claims to British citizenship through a range of political vernaculars, and ‘disoriented ways of thinking about who or what constituted belonging in the British nation’.^22^ This article argues that the entitlements of black Britons to healthcare resources were articulated by healthcare workers, often black women, and that these entitlements had to be articulated in ways not required for the needs of non-minoritized populations. These actors pressured the state to act by making SCA visible as a health condition, employing genetic screening, health education, elevating and leveraging patient testimonies, and drawing on the funding infrastructure of ‘race relations’.

Here I explore the contested health entitlements of black Britons in the NHS by focusing on two moments of contact between people with SCA and the health service—treatment of acute painful sickle cell ‘crisis’ and the debate over community screening initiatives. The article draws on a mixture of archival sources from the period—published research, activist literature, and government correspondence—and also retrospective accounts, such as memoirs and recent oral history interviews. Retrospective sources, particularly from healthcare professionals constrained at the time from denouncing colleagues or criticizing the health service, have been essential for shedding light on the interpersonal dynamics, private meetings and overheard conversations that escape the paper archive of the National Health Service.

## Treating the Crisis: Authority, Pain and the Patient Voice on the NHS ward, 1970s–1990s

In the accounts of the healthcare professionals who pioneered SCD treatment from the 1970s onwards, their first encounters with SCD were often with a patient in intense, unexplained pain. Milica Brozovič, a Yugoslavian haematologist, came to Central Middlesex Hospital in 1975 hoping to conduct research on her particular area of interest—thrombosis. Central Middlesex Hospital was a district general hospital, based in Harlesden in the borough of Brent—a North West London borough which had, in the 1981 census, the highest proportion of people born in the ‘New Commonwealth and Pakistan’ of any council in Britain.^23^ Brozovič recalled that it quickly became clear that thrombosis ‘was not the problem in Central Middlesex, the problem was sickle cell disease’. In her account, this realization crystallized in a meeting with Francis, a young black man with SCD:

[H]e was lying by the bed, swearing, groaning, moaning, crying, making a terrible rumpus. They said, ‘This is your sickle cell disease patient, he’s a druggie, he is a malingerer, that’s what you have’. And then I, being who I am, talking too much, I went to talk to Francis, and discovered that he was absolutely desperate, hating everyone, because nobody believed him he was in pain, so once we gave him painkillers, rehydrated him, he became quite reasonable, and you know I started reading about it, because nobody seemed to know very much about it at that stage at least in Britain.^24^

Francis was experiencing a sickle cell ‘crisis’, caused by blood cells becoming hard and rigid, preventing their easy passage through veins, damaging tissue and organs, and disrupting blood flow in bone marrow. People with SCD commonly describe the resulting pain as ‘excruciating’, and it has been compared to the pain of a heart attack or cancer. In the period under discussion, it was the most common reason for SCD patient admission: between 1962 and 1979, 74 per cent of all SCD admissions in Brent were due to painful crises.^25^ Despite the agony it caused, in 1980, a survey of nurses and health visitors in Brent and in Kensington, Chelsea and Westminster (two of the most diverse boroughs in London) found that only 14 per cent could identify analgesics for pain relief as one of the treatments for SCD.^26^ This was unsurprising given the availability of training and protocols for SCD—no national clinical standards for SCD treatment existed until 2006, and it was rarely referred to in medical textbooks.^27^ This lack of understanding among doctors and nurses about SCD, coupled with paternalist attitudes towards patients in general and stereotyped perceptions of black patients in particular, coalesced dangerously in the event of a sickle cell ‘crisis’.^28^

Brozovič began reading the existing literature on SCD and to give lunchtime talks to nurses, ‘trying to get them to be more sympathetic towards the patients’ and explaining that ‘the pain was a very real thing’.^29^ It was at one such talk that she met Elizabeth Anionwu, an Irish-Nigerian nurse working as a Community Nurse Tutor, who had also taken an interest in haemoglobinopathies. The pair formed a working partnership to address the lack of awareness and inadequate services for this condition in North London. They joined a loose network of people, healthcare professionals and people with SCD across the country, who were organizing around the absence of services for the condition.^30^ As they undertook a number of detailed patient experience surveys, standoffs over pain relief in sickle cell crises emerged as highly charged, racialized and sometimes fatal encounters.

In the accounts of people living with SCD, the same experiences surfaced again and again: after the onset of pain, the person experiencing the crisis would try to ride it out at home, and would resort to hospital when the pain became unbearable. Upon arriving at A&E, they would wait to see a doctor, often for hours. A Brent sufferer recalled waiting four and a half hours without analgesia to see a doctor, and ‘constantly being told off for all the noise that I was making. I felt guilty enough without being told; I didn’t want to keep everyone awake. After about two hours I was moved to another bay to keep some other patients awake’. By morning, he had a temperature of 39.5 degrees centigrade and was, in his words, ‘delirious. Nothing else existed except the agony I was experiencing’.^31^ Helen* described her treatment in the 1980s as ‘a nightmare’ in which she was commanded to restrain her cries of pain. ‘There’s other people to consider, there’s other patients on this ward, you’re not the only patient,’ she recalled being told. ‘You’ve had a painkiller… if it hasn’t worked, then you know, you just want more painkillers’.^32^ Helen’s final sentence points to a common experience described by almost every patient I interviewed and in many patient surveys from the 1980s—the suspicion of the treating nurse or physician that the patient was a drug addict. Suzanne*, who has SCD, was also a doctor who glimpsed this dynamic from the other side. During her haematology rotation in 1984, Suzanne often overheard ‘staff room talk’ about patients on the ward with SCD. ‘Some doctors were really caring’, she said, ‘but other doctors said “oh, they must be addicts. They’re just coming in for morphine, pain can’t be that bad”’.^33^

This neglect meant that some patients were left in agony for hours, and this could be fatal. Suzanne witnessed several children in crises die in waiting rooms because the severity of their condition was underestimated. She remembered ‘one family, she’d lost three children. And they’d all died in the A&E while waiting to be seen, because they had a massive pulmonary crisis, just keeled over’.^34^ Lola Oni, now a specialist nurse consultant, then working as a specialist health visitor in Lambeth, recalled the death of a fifteen-year-old girl as a result of a sickle cell crisis in a south London hospital in the late 1980s, which she attributed to a mistaken belief on the part of the nurses that a sickle cell crisis could not be fatal.

[S]he’d been calling out for the nurses, wanting pain relief because she was in so much pain, because sickle pain, you can’t see it… here’s a young girl, black young girl demanding opiates. Eventually she calmed down and the nurses were so grateful, ‘oh she’s gone to sleep, thank goodness’. And so they did the medicine round… she was very sleepy… They put the glass to her mouth. She took her medication, and they went away. A couple of hours later, they were doing the observation round, and she was dead. She’d had a bleed in her brain, that’s why she’d gone to sleep… the shock reverberated down the ward. The nurses were, there were tears.^35^

This girl’s pain had been invalid when she asked for pain relief because, as Lola observed, she was ‘a black young girl demanding opiates’. In the light of these accounts, the death of Stephen Bogle appears not as a result of police incursion into welfare state territory. Bogle’s experience in the last days of his life actually mirrored those of some SCD patients on the wards of NHS hospitals, at the hands not of police officers, but of healthcare professionals. Kennetta Hammond Perry has argued that the intersection of the carceral and welfare states in Britain facilitated rather than mitigated state violence.^36^ Bogle’s poor treatment was not just indicative of the institutionalized racism of the Metropolitan Police, but of a broader racialized pattern of exclusion from the social rights of citizenship as a whole, perpetrated by the caring as well as the controlling arms of the state.

This accusation of drug addiction was, in many cases, racialized, though access to pain relief could be inconsistent for patients across the board. During the 1980s there was considerable medical anxiety about the highly addictive painkiller pethidine, which was commonly used to treat sickle cell pain.^37^ Pain relief for patients in NHS hospitals could be poor: a 1977 survey in The London Hospital found that few patients were free of pain and many with malignant or terminal illnesses did not know their diagnoses.^38^ One doctor at a symposium about SCD pain relief in hospitals reflected that ‘when I had my gall bladder out and lay in a surgical ward for a few days it became apparent to me that both doctors and nurses are remarkably bad at treating acute pain, not just in sickle cell disease but post-operative pain and I think in other sorts of acute pain too’.^39^

But, unlike in these general cases, patients with sickle cell disease frequently reported that nurses or doctors assumed them to be already addicted to drugs (rather than at risk of addiction), and found their pain ascribed not to their biology but to social factors. These accusations of drug addiction drew on wider state and societal stereotyping of black communities. Accusations of drug activity were often cited by police operations when raiding popular black businesses or social spaces (most famously, the repeated police raids on the Mangrove restaurant in 1969 and 1970), and there were instances in which police officers exploited these racist stereotypes by threatening to plant drugs on black men deemed to be ‘uncooperative’.^40^ As Mike Brake and Gregory Shank argued in the early 1980s, black people were widely perceived as ‘living first off immoral earnings, then by defrauding welfare, drug pushing, and finally mugging’, despite statistical evidence to the contrary.^41^ The accusation of drug seeking levelled at SCD patients combined the racist stereotypes of defrauding the welfare state and of drug possession. In the realm of law enforcement, drug activity was cited as a justification for police harassment—in medical spaces, drug use or dependence could be used as a rationale for the withdrawal of medical care.

Racist stereotypes about pain thresholds and the expression of pain may also have played a part in the under-treatment of SCD pain. Elizabeth Anionwu and Karl Atkin noted that ethnic minority patients were frequently perceived as ‘being trivial complainers’ and ‘time wasters’, and that there was a widespread belief in the ‘lower pain thresholds’ of African Caribbean people.^42^ Rob Boddice argues that structures of racism can be enforced through conceptual notions of ‘emotional superiority’, in which the oppressor prescribes ‘emotional conformity, making for extremely strict emotional regimes that challenged those deemed racially inferior to meet almost impossible standards of emotional expression, given the often harsh or torturous conditions in which the emotives took place’.^43^ The rule implicit in these encounters—‘you’ve had a painkiller, if it hasn’t worked then you just want more painkillers’ and ‘they’re just coming in for morphine’—meant that every effort to articulate pain was only read by medical staff as ‘proof’ of drug-seeking or of cultural stereotypes about pain thresholds.^44^ Sara Ahmed has argued that the figure of the ‘bogus asylum seeker’, gaining access to the benefits of British residence through deception, is an entrenched hate figure in British political discourse which ‘justifies our intrusion into the bodies of others’.^45^ Within the NHS, young SCD patients were dismissed as ‘bogus’ patients, faking illness to gain access to state-funded medical attention, and justifying medical skepticism and scrutiny.

As clinics, haematologists and nurses specializing in SCD emerged in the 1970s and early 1980s, some began to act as intermediaries between patients, their treating physicians, and the broader edifice of the NHS. In seeking to address poor care for sickle cell crises, interested healthcare professionals conducted patient surveys, or drew upon the work of patient organizations, to support urgent recommendations. Voluntary groups such as the Organization for Sickle Cell Anaemia Research (founded by patient activist Neville Clare), the Sickle Cell Society (co-founded by Anionwu) and other groups such as the Runnymede Trust, to produce sometimes damning collective narratives of SCD patient experience within the NHS.^46^ These publications positioned people with SCD within the burgeoning patient–consumer movement. Interest groups championing patient and consumer rights had gathered steam from the 1960s onwards, and had touched on issues of discrimination against ethnic minorities.^47^ Anti-racist campaigners had focused on consumer rights—the Campaign Against Racial Discrimination (CARD) had devoted some of its efforts in the 1960s and 1970s to legislating for b lack Britons’ equitable access to the consumer market.^48^ Such movements, tying the rights of British citizenship to consumer rights, were representative of ‘the elision of social democratic citizenship with consumerist selfhood’ in the figure of the ‘citizen-consumer’, which had emerged in the decade following the post-war settlement.^49^

Within the published SCD surveys, the testimonies of SCD patients were sometimes explicitly connected to British citizenship—at a 1983 symposium on SCD pain, Elizabeth Anionwu played an extract from a taped interview she had conducted with a 17-year-old girl in 1980. The girl expressed that her doctors did not understand her condition, nor know how to treat the pain she lived with constantly, and concluded ‘I suppose I’ve got to accept what they give, being in their country’.^50^ The ‘seriousness’ of this statement, Anionwu said, had led to a reappraisal of the services for SCD at the hospital. Playing these words aloud to a room of healthcare professionals, Anionwu showed that the lack of provision for SCD communicated to patients that they did not belong, and that this failure of black patient-consumers must be put right.

Such patient criticisms of the service were increasingly picked up in medical literature, brought to the attention of the wider medical community and cited in research into best practice for treatment for years.^51^ In 1987, Brozovič and her colleagues argued in the British Medical Journal that it was ‘essential’ that guidelines for the management of SCD ‘are formulated, published and implemented along the lines of the Department of Health guidelines for haemophilia and the US guidelines for sickle cell disease’.^52^ In 1993, the paediatric haematologist Sally Davies (who would be Chief Medical Officer from 2010 to 2019) wrote an opinion piece for the *BMJ* calling for urgent research into pain relief for sickle cell crises.^53^

These health professionals also attempted to educate or confront colleagues in their workplaces or local health authority. Lola Oni, a Nigerian-born health visitor, was one such professional. She began work at the Lambeth Sickle Cell and Thalassaemia Centre in 1985, and a critical focus of her work over the following eleven years was to legitimize the pain of sickle cell crisis through face-to-face re-education of medical personnel. She delivered workshops and accredited courses, and ran case scenario group work across London hospitals, in order to improve understanding of the clinical standards and protocols needed to treat sickle cell disease. Oni recalled that the doctors and nurses they spoke to ‘were receptive to the education, but that didn’t change attitude. This is what we discovered—that despite all the education we did, and people understood, attitudes did not change’. She attributed this to the racist notion of young black people as ‘junkies’, and said the attitude of ‘utmost disdain’ from nurses was difficult to shift. But Oni stressed that how a sickle cell patient would be treated in a ward was often a reflection of the attitude of the ward sister.

If you have a ward sister that believes that all sickle patients are junkies and a pain in the neck, you'll find that most of the staff will feel that way too, because they will want to be accepted by the ward sister… [at the hospital I worked at] we had a ward sister that was very – I'm not even sure it was it was racism or wickedness… I couldn't understand why a lot of the staff were just so resistant to teaching, but when that ward sister left, the ward changed dramatically.^54^

Structure and authority within a hospital ward could enforce an emotional regime in which SCD pain was invalid. In a work environment in which black nurses were often much less likely to gain promotions and to occupy more senior nursing positions, this could have far-reaching consequences—though in such ward environments, Oni said, ‘black nurses were not necessarily sympathetic’ either.^55^

Oni’s analysis demonstrates that the diversity of the NHS workforce could not be an automatic salve against the structural racism of the service. Her words attest to the difficult circumstances that black NHS workers had to navigate—employed in a structurally racist system, witness to the racist treatment of black patients, while themselves facing professional discrimination and precarity. Into the 1980s and 1990s, the emerging profession of sickle cell nurse counsellors—most of them black women—were the key professional figures in sickle cell policymaking, and formed a professional body, the Sickle Cell and Thalassaemia Association of Counsellors (STAC). As services sprang up in London, Manchester, Liverpool, Leeds, Leicester, Nottingham, Birmingham and beyond, these specialist nurse counsellors ‘were consistently regarded by families [with sickle cell] as their key worker and are the most praised of all professional contacts’, although the precarious funding of these services often meant that they were underpaid for their work.^56^ In 1985 Stella Dadzie, Suzanne Scafe and Beverley Bryan pointed to the role black women played in informing their communities about SCD and raising funds for research and services. They were more circumspect about the emergence of ‘black health advisors’ within the NHS, warning of ‘the dangers of ghettoizing black health concerns within the NHS’ and skeptical that the NHS could ever be reformed from within.^57^ But many of these black healthcare professionals recognized the limitations of the hierarchical NHS structure for accountability, and adapted by working across both the health service and voluntary organizations. Catherine, a black paediatrician, used the metaphor of taking off and putting on different ‘hats’—her ‘medical’ hat, and her ‘charity’ hat—when discussing her SCD advocacy in the 1980s. Her efforts to raise awareness and attract funding for the condition through her professional channels initially gained little traction. But through supporting sickle cell charities by providing medical advice, and speaking as a charity representative, she said, ‘[I] put my professionalism on one side… and [was] a pain in the, a thorn in the side of the authorities’.^58^ In this way, Catherine and others like her were able to challenge the ‘collegial’ model of medical self-regulation in the 1980s NHS, which could bring ‘devastating professional consequences’ to doctors and nurses who spoke up.^59^

The example of sickle cell crisis, pain and its treatment illustrates two things. First, that people experiencing SCD crisis were frequently, sometimes fatally, read by many healthcare professionals as social problems, afflicted with drug addiction, and the reality of their illness disbelieved.^60^ Drawing on societal and medical racism, Black British patients were framed as ineligible for health care. Secondly, this example shows that the meaning of the sickle cell crisis was contested on the wards of the NHS, as healthcare professionals confronted their peers with patient testimonies, attempting to communicate the difficult position of a patient in pain, and stress the seriousness of sickle cell crisis. With the advent of the NHS internal market in 1990, framed as the most effective way of delivering quality care to patient-consumers, SCD patient activists and their healthcare worker allies worked to incorporate sickle cell, and anti-racist critique, into the move towards consumerism within the NHS.^61^ In 1993, the Department of Health launched a range of ‘Patient Perception Booklets’, aimed at purchasers to ‘help them with contracting for a high quality, patient responsive service’. One such booklet, authored by Anionwu, was entitled ‘Sickle Cell Disorder: Patient’s perceptions of coping with pain’ and contained personal testimonies from patients articulating the emotional impact of delays in pain relief, though it stopped short of explicitly discussing racism as a factor in medical care.^62^ Adapting to a shifting lexicon of British welfare citizenship, healthcare workers positioned Black British patients as underserved patient-consumers.

## Hidden in Plain Sight—Local Health Authorities and Resources for SCA Screening and Treatment

The primary intervention considered for genetic conditions in the 1970s and 1980s was genetic screening—either to catch a genetic condition early so that it could be treated, to diagnose a fetus with the condition so that parents could decide whether to continue with the pregnancy, or to screen individuals so that they could be informed about their risk of having a child with a genetic condition.^63^ However, local health services considered SCA to be an intrinsically foreign condition, not present in Britain in sufficient numbers to justify the expense. This logic existed even in areas heavily policed for their high ‘immigrant populations’, and was reinforced by the resulting absence of screening pilots and surveys to gauge the rates of the SCA trait in these areas. Local health professionals who petitioned their Local and Regional Health Authorities for the resources to provide community screening for SCA found their requests refused on the grounds of two main objections: first, that the local areas were not diverse enough for the intervention to be cost effective; and second, that black families were not suitable for screening for social and cultural reasons. This illustrates the parallel geographies of the welfare state, in which black people could be hyper-visible to state actors invested in social services and public order, but invisible to those managing the resources of local public health.

Roberta Bivins has shown that post-war British medical and state institutions were keen to inscribe SCA with a ‘tropical identity’ by locating it firmly beyond British shores, reflecting successive governments’ ‘extreme ambivalence about the permanent presence within Britain of South Asian and West Indian ethnic populations’.^64^ British medical texts from the 1960s and 1970s preserved the notion that SCA did not affect British people. An understanding of Britain as a ‘white country’, and SCA’s racialization as a primarily ‘African’ or non-white trait precluded researchers, doctors and policymakers from considering it relevant. Healthcare professionals and experts engaging with the condition geographically delimited the condition to Africa, India, the Middle East and the Caribbean. One 1964 film, ‘Sickle Cell Anaemia in Nigeria’, produced for the education of British doctors, left North America and Britain blank on a map showing the incidence of sickle cell trait, even as the narrator euphemistically explained that the trait could be found ‘wherever men of African descent have migrated’.^65^ By the time this film was made, Commonwealth migration to the British metropole (from many of these shaded regions) had been underway for over fifteen years with an estimated 900,000 migrants from former colonies residing in Britain. Of course, African-American populations in the USA had been present for centuries.^66^ Twelve years later, the 1976 edition of the key medical textbook *Genetic Counselling* still claimed that SCA ‘is not of great consequence to us in the context of genetic counselling in the United Kingdom’, because the condition is ‘confined to peoples of African and Eastern origin’.^67^ This racialized and geographical understanding of SCA rendered the trait and disease largely invisible in Britain until the 1980s, and hindered the development of treatments and services. Such approaches constituted a denial, on a population level, of the citizenship status of Commonwealth migrants to Britain and their families.

Despite the widely-accepted notion that sickle cell affected primarily African, Caribbean and (to a lesser extent) Asian and Mediterranean people, advocates for SCA screening struggled to convince Health Authorities to undertake pilot screening programmes even in Britain’s most diverse areas. A 1971–75 survey of live births in London boroughs had shown that in Brent (which is included in this particular health authority) 38 per cent of live births were born to mothers from the New Commonwealth and Pakistan—the highest in London.^68^ Yet even by 1981, Milica Brozovič asked the North West Thames Regional Health Authority for funds for a selective screening programme, and recalled that the Authority refused, saying ‘we do not have that many [people from ethnic minorities], it is not cost effective to do it’.^69^ Given the high number of black and Asian people in Brent, if any borough in London (and therefore probably in Britain) had sufficient numbers for a cost-effective SCD screening pilot, it was Brent. The indifference of the health authorities in Brent contrasted with the attitude of local policing in the Brent area—in 1971, a National Opinion Poll survey into race relations had noted that ‘West Indians in Brent were particularly critical in thinking that the police generally pick on coloured people and did not deal with them fairly in their locality’.^70^ Brozovič and her colleagues focused on proving that ‘we did indeed have that many, that we had more than Tay Sachs… more than Down’s Syndrome’, and undertook a pilot screening programme at the Central Middlesex maternity unit. The estimated cost was £20,000, which Brozovič took from their existing budget, remembering they had to ‘squeeze here, squeeze there, but it was a hard battle’.^71^ Catherine, a community paediatrician who was herself black, had a similar experience, when she asked her Local Health Authority (LHA) to trial newborn screening for SCA when she was based at a hospital in Paddington.^72^ The LHA were so reluctant that Catherine and her manager cut costs by volunteering out of hours to conduct genetic counselling and outpatient follow-up, and through performing only selective screening focused on ethnic minority mothers. Such selective screening programmes were less effective than universal screening, Catherine explained, as skin colour was not an accurate predictor for SCA, because it relied on healthcare professionals making uninformed judgements about a mother’s ethnic background, and because she had encountered self-described White British people who tested positive for the sickle cell trait.^73^ Healthcare professionals pragmatically leaned in to notions of SCA as an ethnic minority trait, volunteered their time and reworked their existing budgets to prove that there was a need for screening in their local areas.

Even where it was conceded by LHAs that there was an ‘immigrant’ population of an appropriate size to begin or extend SCD screening, objections were still raised based on the amenability of black communities to genetic intervention. These screening programmes were often limited to pregnant mothers (excluding fathers), looking to identify cases of SCD in newborns rather than the asymptomatic sickle cell trait. Health Authorities and health professionals expressed reluctance to extend the screening programme to pregnant women’s partners, and were pessimistic about informing families if their child had the asymptomatic trait. Anionwu recalled a meeting initiated by the Camden & Islington Health Authority in June 1981. Despite their enthusiasm in setting up the meeting, Anionwu writes that it quickly became clear that the officials considered local services for SCA to be already ‘adequate’. They had a screening programme for pregnant women who were black or Asian, but argued that it was ‘impractical to extend the screening to the partners’—despite the fact that University College Hospital did have partner screening for women with thalassaemia trait, considered more common in Mediterranean families. Anionwu concluded that there was ‘an underlying racist and patronizing attitude that influenced the response of Authority members’, who had made assumptions that

the black community were not educated enough to understand genetics and the high number of single black mothers would make it difficult to trace fathers. There was an insinuation that this group was promiscuous and lacked stable relationships simply because they were not married, revealing a disturbing degree of ethnocentrism and failure to understand lifestyles different from their own white, male, middle class one.^74^

These two objections—intellectual grasp of genetics and uncertain paternity—were frequently cited by Health Authorities and researchers. A Manchester neonatal screening pilot, set up also to test pregnant women and their partners, did not test many of the fathers ‘because the mothers were not married’.^75^ Such insinuations of promiscuity and instability in black families were continuations of 1950s liberal anxieties that ‘British home life seemed impenetrable to migrants of colours’.^76^ These endured in what Tracey Reynolds has described as ‘myth making and moral panic’ around black families that eschewed the mold of the ‘nuclear family’ in public debate, research and policymaking in Britain.^77^ Health service planners and commissioners viewed the clinical needs of Black British families through a social and moral lens, invalidating their entitlement to public health services.

Some medical professionals were pessimistic about the suitability of migrant families for screening interventions. The author of the Manchester screening pilot, one David Evans, wrote to the *British Medical Journal* in 1974 rebutting an editorial arguing for haemoglobinopathy screening, arguing that such a programme would be pointless when the target population had little understanding of SCD:

Parents cannot understand the genetic implications of being carriers if they know nothing about the disease in question. If a programme of screening and education is to have a chance of success it must be run by black people and the impetus should come from the black community, but there are few individuals who could organize an educational programme in the black community here.^78^

Evans’s anxieties were possibly linked to doctors’ concerns about discussing what they saw as a ‘black disease’. Patient activist Neville Clare reported that when he made contact with doctors at Kings College Hospital in 1975, they had not conducted outreach with the local community in Brixton because ‘they knew that if they alone went to Brixton… and started to talk about the facts of sickle cell, the reaction would be uniformly hostile’. Clare himself reported that his first educational talk about sickle cell, at the West Indian Student Centre in 1976, divided the audience—with some arguing that SCA was ‘a concoction of the white man, part of their plan to discredit the black race’.^79^ The weaponization of SCA by the National Front, combined with works of scientific racism on race and IQ in the 1970s, meant that discussion of ethnicity-linked disease was highly charged.^80^ These medical concerns about backlash to such outreach were combined with negative assumptions about black community work—with Evans concluding that ‘few individuals… could organise an educational programme in the black community here’. As such, infantilizing assumptions about the black community existed not in opposition to, but (to borrow Stuart Hall’s phrase) ‘cozily inserted within’ liberal concerns about the appearance of racism.^81^

Five years after Evans’s intervention, Elizabeth Anionwu and Milica Brozovič established the Brent Sickle Cell and Thalassaemia Centre—the first of its kind in Britain—in 1979. Its services included walk-in screening, genetic counselling, bereavement counselling, and home and ward visits to patients. By 1985, the centre covered ‘an estimated Brent population of 36,000 Afro-Caribbeans, as well as people from adjacent districts’, and had picked up 193 recorded cases of SCD in the borough—increasing to 300 cases by 1987 and to over 400 by 1993.^82^ In the years following the establishment of the Brent centre, a number of centres were set-up across England and Wales, and by 1985 seven such centres existed.^83^ These centres, such as the sickle cell centre set-up in Manchester’s Moss Side in 1984, were the result of campaigning by local sickle cell support groups, Community Health Councils, interested healthcare professionals and voluntary organizations. The same period saw the emergence of clinical nurse specialists, trained in providing information and genetic counselling about haemoglobinopathies.^84^ However, despite the British Society for Haematology’s 1988 recommendation that ‘[n]eonates from *at-risk* communities must be screened at birth for SCD… it may be necessary in some districts to screen all babies’, even boroughs with high percentages of at-risk populations lacked such services.^85^ In 1991, Wandsworth had ‘the second highest proportion of Afro-Caribbean people of any London borough’ but no SCD centres, nor universal neonatal screening. Wandsworth health visitor Faye Harry remarked that ‘Dr Sherman at St George’s Hospital holds regular seminars on sickle cell, but for the life of her she can’t get any money to set up a sickle cell centre here’, which meant that families needing support and advice had to travel to a unit in Camberwell.^86^

Most of these centres were funded by a combination of short-term funds, from health authorities, councils, and very often from a scheme called Inner City Partnerships (ICPs), formerly known as the Urban Programme. The Urban Programme, founded in 1968, had been originally earmarked as funds to address the social problems of England’s ‘inner cities’—a euphemism, Otto Saumarez Smith has argued, that served as a ‘semi-covert way to address issues of new immigrant communities’ without arousing the resentment in the white British population.^87^ This initiative gained a new lease of life just as SCD reached Westminster’s agenda, both as a result of the 1980–81 uprisings. In 1980, the Home Affairs Committee on Racial Disadvantage had heard evidence concerning insufficient screening for SCD, and recommended to the Department of Health and Social Services (DHSS) that ‘hospitals in high-risk areas should consider providing neonatal and adult screening facilities’.^88^ After the uprisings which swept across 26 cities in Britain from April to July 1981, the budget of the Urban Programme/ICPs was increased to £270 million for 1982/83, and 200 new ‘ethnic projects’ were approved, overseen by the Department of Environment (DoE).^89^ DHSS encouraged health authorities to apply to the programme, and the following year, DHSS reported back to the Home Affairs Committee that ICP funding had been approved for SCD screening and counselling services in Brixton and Hackney in London, with plans to expand elsewhere.^90^ By 1985, of the seven dedicated sickle cell centres in England, four were supported by short-term Inner City Partnership funding—Liverpool, Lambeth, Brent and City & Hackney.^91^

Given that local healthcare professionals had struggled to persuade their LHAs to provide funds from their existing budgets, it is unsurprising that so many sickle cell centres turned to the Department of Environment’s ICP funds. It was a pragmatic, short-term solution that allowed them to pilot sickle cell centres in areas with a concentrated group of those ‘at risk’, where the necessary expertise could be generated.^92^ In response to the availability of this funding, LHAs and healthcare professionals styled SCA as a natural feature in the ecosystem of urban health—perceived not as a medical problem, but a social one. Other projects related to ethnic minority health funded by the ICP included illnesses caused by nutritional deficiency, such as rickets; ‘the effects on health of child rearing practices in West Indians which differ substantially from the norm in Britain’; and ‘the failure of ethnic minority groups to use NHS services’.^93^ As such, health issues of ethnic minorities were attributed to genetic difference, cultural practice, or their ‘failure’ to use NHS services, rather than an absence of funding or interest from the state, or structural issues of poverty and discrimination. Some contemporaries had warned that this would be an outcome of the Urban Programme. Ambalavaner Sivanandan had argued that ‘instead of actually addressing racial injustice, the programme redefined the problem as one of cultural disadvantage and went on to reinforce cultural differences without making any changes to a discriminatory system’.^94^

The reluctance of the National Health Service, and DHSS, to implement a national screening programme for haemoglobinopathies was not unusual. Internationally, post-war governments were reluctant to mandate genetic screening programmes.^95^ Moreover, screening and treatment was implemented in piecemeal form across many health conditions, from cervical cancer to tuberculosis, by Local Health Authorities during the period.^96^ But while the poor treatment for SCD in much of the NHS was (in some ways) unexceptional for rare health conditions, this article has sought to show that the services that emerged around screening, diagnosis and treatment on the ground were highly racialized. People with the sickle cell trait or disease were constructed as a geographical, cultural and social minority, ineligible for NHS care—and such approaches implicitly reified Britain as a ‘white’ country. As Roberta Bivins has argued, ‘public health’ shifted in the post-antibiotic period from ‘large-scale centrally directed interventions’ to ‘individual idiosyncrasy, whether genetic, biochemical or “cultural”’, with inconsistent results for migrant communities.^97^ The case study of SCD illustrates that, even in a health system increasingly attuned to idiosyncrasy, perceived differences to an imagined British ‘public’ often disqualified black communities from the services they needed.

Genetic screening programmes are a method of making the unseen seen—using accurate numbers of those living with the condition, services can be lobbied for and designed to meet need. Without these screening programmes, the true size of the SCD population was uncertain, and cases may have been missed.^98^ As Usha Prashar argued in 1985, this ‘absence of statistics is in turn taken to represent the absence of individuals affected and used as an excuse for the failure to provide suitable facilities for screening and counselling’.^99^ Activist healthcare workers, through their pilot screening programmes and sickle cell centres, developed a body of expertise and evidence that eventually culminated in the implementation of a national screening programme for sickle cell in 2005. Following this, the number of children identified with SCD dramatically increased, in some areas doubling the pre-existing workload. In assessing the results from the first two years of the screening programme, the programme team concluded that ‘[u]nderascertainment of the condition has allowed a downplaying of the scale of need’ and may have factored into higher infant mortality rates in cities ‘as babies died without a diagnosis or treatment’.^100^ The invisibility of these patients hindered the case for interventions such as screening and counselling, and the limited data available on patient numbers also enabled the NHS to avoid accountability for these absent services.

## Conclusion

Both an ambulance and a police car were sent to collect Stephen Bogle from the Hackney chip shop on that summer’s day in 1986, and the Sickle Cell Society identified his entrance into police custody as one of ‘a number of occasions… when an opportunity was lost for ensuring that he received all proper treatment for his condition’.^101^ Bogle’s case makes plain the ways in which physical illness in Black British citizens could be construed by state actors as a cover for criminality. However, the accounts of being treated for sickle cell crisis on the wards of the NHS demonstrate that Bogle’s death was not an isolated incident precipitated by police indifference, but was symptomatic of how black people and their physical health were perceived and treated within the British welfare state, and within the spaces of the NHS. This treatment continues logics embedded in longer histories of the health service in Britain, imagined as a reward for a whitewashed wartime sacrifice, and made possible by the labour of Commonwealth workers both in former colonies and in the metropole. In this institution, black people were constructed and perceived, on an individual and population level, as too expensive or difficult to treat. This article has focused on SCA as a case study that reveals these broader dynamics, and it was seen as such in the 1980s. Suzanne Scafe, Stella Dadzie and Beverley Bryan called the NHS ‘the uncaring arm of the state’, arguing that it was an institution which saw black people, and particularly black women, as workers for, but not consumers of, the health service.^102^ The black health activist Allan McNaught contended in 1987 that although SCA was not the primary health concern of ethnic minorities, it was useful as a benchmark ‘to assess the willingness and ability of the NHS to respond to the specific needs of ethnic minorities’.^103^

What was at stake in these discussions over SCA was the wider question of whether ‘black health’ was ‘British health’. In an exchange in the Sickle Cell Society newsletter in 1993, the footballer Garth Crooks wrote that ‘Sickle cell is a British problem affecting British people’, echoed the following month by Health Secretary Baroness Cumberlege, who remarked that it ‘caught my attention’.^104^ Within the NHS, health workers pushed to make black people and their health needs visible and undeniable to the state. To do this they deployed various lexicons of citizenship, from notions of patient-consumership to population surveys; and different pragmatic strategies, from reallocating their own NHS budgets, to leaning into state notions of urban health and selective screening. In doing so, they contested whitewashed citizenship and welfare state entitlements, claiming a space for black people as consumers of the health service, whose needs must be met.

## Footnotes

1 Black Cultural Archives (BCA), Brixton, RC/RF/17/02/C, ‘Note on Inquest on Stephen Bogle (sickle cell patient) who died at Thames Magistrates Court on 12 August 1986’, 20 January 1987.

2 Letter, Douglas Hurd to David Steel, 13 April 1987, BCA RC/RF/17/02/C.

3 Institute of Race Relations, *Deadly Silence: Black Deaths in Custody* (London, 1991): 20.

4 Sickle Cell Society, ‘Note on inquest on Stephen Bogle’, BCA RC/RF/17/02/C.

5 Though a Metropolitan Police Complaints Investigation Bureau inquiry was conducted, the report and its conclusions have not been made public. I made a Freedom of Information request to the Metropolitan Police Service (MPS) for the report of this investigation in March 2018. The MPS deemed the 400-page report exempt, citing Section 38 (that the document if released could damage an individual’s health and safety—in this case Bogle’s family), which was upheld by the Information Commissioners Office. For more on how the use of personal exemptions can also obscure the operation of the state, see Jordanna Bailkin, *The Afterlife of Empire* (Berkeley, 2012), 13.

6 Works that have discussed the racial politics of sickle cell anaemia in Britain and the USA include Simon Dyson, *Sickle Cell and the Social Sciences: Health, Racism and Disablement* (Abingdon, 2019); Roberta Bivins, *Contagious Communities: Medicine, Migration and the NHS in Post-war Britain* (Oxford, 2015), 304–67; Elizabeth Anionwu and Karl Atkin, *The Politics of Sickle Cell and Thalassaemia* (London, 2001); Melbourne Tapper, *In the Blood: Sickle Cell Anaemia and the Politics of Race* (University of Pennsylvania Press, 1999); Keith Wailoo, *Dying in the City of the Blues: Sickle Cell Anemia and the Politics of Race and Health* (Chapel Hill, 2001); Carolyn Moxley Rouse, *Uncertain Suffering: Racial Health Care Disparities and Sickle Cell Disease* (Berkeley, 2009). I follow Melbourne Tapper in referring to sickle cell anaemia (SCA) as an umbrella term for both the trait and the disease. I will specify when I am referring particularly to either sickle cell disease (SCD) or the sickle cell trait. See Melbourne Tapper, ‘Interrogating Bodies: Medico-Racial Knowledge, Politics and the Study of a Disease’, *Comparative Studies in Society and History* 37 (1995), 76–93.

7 Matthew Grant, ‘Historicizing Citizenship in Post-War Britain’, *The Historical Journal* 59 (2016), 1193.

8 James C. Scott, *Seeing Like a State: How Certain Schemes to Improve the Human Condition Have Failed* (New Haven and London, 1998), 11.

9 Roberta Bivins, ‘Immigration, Ethnicity and “Public” Health Policy in Postcolonial Britain’, in Catherine Cox and Hilary Marland, ed., *Migration, Health and Ethnicity in the Modern World* (Basingstoke, 2013), 126–50.

10 Robbie Shilliam, *Race and the Undeserving Poor: From Abolition to Brexit* (New York, 2018).

11 Camilla Schofield, *Enoch Powell and the Making of Postcolonial Britain* (Cambridge, 2013), 15.

12 Gary Craig, ‘“Cunning, Unprincipled, Loathsome”: The Racist Tail Wags the Welfare Dog’, *Journal of Social Policy*, 36 (2007), 612; Elizabeth Buettner, ‘‘This is Staffordshire not Alabama’: Racial Geographies of Commonwealth Immigration in Early 1960s Britain’, *Journal of Imperial and Commonwealth History*, 42 (2014), 716.

13 Paul Gilroy, *‘There Ain’t no Black in the Union Jack’: The Cultural Politics of Race and Nation* (London, 2002), xxxiii.

14 Bivins, *Contagious Communities*, 13.

15 Bailkin, *The Afterlife of Empire*, 24.

16 Christopher Kyriakides and Satnam Virdee, ‘Migrant Labour, Racism and the British National Health Service’, *Ethnicity and Health*, 8 (2003), 286–7.

17 Nadja Durbach, ‘One British Thing: A Bottle of Welfare Orange Juice, c. 1961 – 1971’, *Journal of British Studies*, 57 (2018), 564–67.

18 Kyriakides and Virdee, ‘Migrant Labour, Racism and the British National Health Service’, 289.

19 Roberta Bivins, ‘Picturing Race in the British National Health Service, 1948-1988’, *Twentieth Century British History* (2017), 19; Kyriakides and Virdee, ‘Migrant Labour, Racism and the British National Health Service’, 292.

20 Julian M. Simpson, *Migrant Architects of the NHS: South Asian Doctors and the Reinvention of British General Practice (1940s-1980s)* (Manchester, 2018).

21 Bivins, ‘Picturing Race in the British National Health Service’, 5.

22 Kennetta Hammond Perry, *London is the Place for Me: Black Britons, Citizenship and the Politics of Race* (Oxford, 2016), 12.

23 Malcolm Cross, Harbajhan Brar and Mike McLeod, *Racial Equality and the Local State: An Evaluation of Policy Implementation in the London Borough of Brent* (Warwick, 1991), 11.

24 Milica Brozovič, interview with author, October 2018.

25 Elizabeth Anionwu, Diana Walford, Milica Brozovič and Betty Kirkwood, ‘Sickle Cell Disease in a British Urban Community’, *British Medical Journal* 282, (24 January 1981): 283.

26 Elizabeth Nneka Anionwu, ‘Health Education and Community Development for Sickle Cell Disorders in Brent,’ PhD dissertation. University of London Institute of Education (1988), 110–12.

27 The standards and guidelines for care of SCD in childhood were launched in 2006 and for adults in 2008. Sickle Cell Society, *Standards for the Clinical Care of Adults with Sickle Cell Disease in the UK* (London, 2008).

28 Anionwu and Atkin, *The Politics of Sickle Cell and Thalassaemia*, 98–99.

29 Milica Brozovič, interview with author, October 2018.

30 For more on this early period of SCD activism, see Neville Clare, *An OSCAR For My Troubles: A Life Working for Better Understanding and Treatment of Sickle Cell Disorder* (Oxford, 2007). Health activist Protasia Torkington undertook action research around poor SCA services in Liverpool in the late 1970s, see Ntombehle Protasia Khotie Torkington, ‘Racism in the National Health Service: A Liverpool Profile’, PhD thesis, University of Liverpool (1985), 186–7.

31 Sickle Cell Society, *Pain in sickle cell disease: ‘I know that you are in pain, but would you mind answering a few questions?’* (National Extension College and Sickle Cell Society, 1986), 67.

32 Helen (pseudonym), interview with author, August 2018.

33 Suzanne (pseudonym), interview with author, March 2018.

34 Suzanne (pseudonym), interview with author, March 2018.

35 Lola Oni, interview with author, April 2018.

36 Kennetta Hammond Perry, ‘The Archive of Black Life and the Architecture of State Power: The Case of David Oluwale’ (unpublished paper) London Group of Historical Geographers Seminar (22 January 2019).

37 Joanna Bourke, *The Story of Pain: From Prayer to Painkillers* (Oxford, 2014), 296.

38 J. M. Hunt, T. D. Stollar, D. W. Littlejohns, R. G. Twycross, and D. W. Vere, ‘Patients with Protracted Pain: A Survey Conducted at the London Hospital’, *Journal of Medical Ethics*, 3 (1977), 61–73.

39 Sickle Cell Society, *Pain in sickle cell disease*, 29.

40 Rob Waters, *Thinking Black: Britain, 1964-1985* (Oakland, 2019), 97; Perry, *London Is The Place For Me*, 235.

41 Mike Brake and Gregory Shank, ‘Under Heavy Manners: A Consideration of Racism, Black Youth Culture, and Crime in Britain’, *Crime and Social Justice* 20 (1983), 6.

42 Elizabeth Anionwu and Karl Atkin, *The Politics of Sickle Cell and Thalassaemia* (Buckingham, 2001), 96.

43 Rob Boddice, *The History of Emotions* (Manchester, 2018): 260.

44 This trope of ‘addict’ SCD patients was not limited to Britain but was also a common feature of SCD treatment in the USA. See Rouse, *Uncertain Suffering*, 71–2.

45 Sara Ahmed, *The Cultural Politics of Emotion* (Abingdon, 2004), 47.

46 Janet Black and Sophie Laws, *Living with sickle cell disease: An Enquiry Into the Need for Health and Social Service Provision for Sickle Cell Sufferers in Newham* (London, 1986).

47 In 1967, the social survey organization Political and Economic Planning (PEP), connected to the organized consumer movement, published a survey demonstrating widespread discrimination against non-white Commonwealth migrants in employment. Alex Mold, ‘Patient Groups and the Construction of the Patient-Consumer in Britain: An Historical Overview’, *Journal of Social Policy* (2010), 506, 509; Erik Bleich, *Race Politics in Britain and France: Ideas and Policymaking since the 1960s* (Cambridge, 2003), 94, 171.

48 Perry, *London is the Place for Me*, 245.

49 Alastair Kefford, ‘Housing the Citizen-consumer in Post-war Britain: The Parker Morris Report, Affluence and the even Briefer Life of Social Democracy’, *Twentieth Century British History*, 29 (2018), 258.

50 Sickle Cell Society, *Pain in Sickle Cell Disease*, 18.

51 M.L. Tillyer, ‘Letter: Services for Sickle Cell Disease’, *British Medical Journal*, 296 (19 March 1988), 865.

52 M. Brozovič, S. C. Davies and A. I. Brownell, ‘Acute Admissions of Patients with Sickle Cell Disease who live in Britain’, *British Medical Journal*, 294 (1987), 1208.

53 Sally C. Davies, ‘The Vaso-occlusive Crisis of Sickle Cell Disease’, *British Medical Journal*, 302 (29 June 1991), 1551–2.

54 Lola Oni, interview with author, 10 April 2018.

55 Sharon Beison, Satnam Virdee and Ann Hagell, *Nursing in a Multi-Ethnic NHS* (London, 1995), 103; Lola Oni, interview with author, 10 April 2018.

56 Simon Dyson, interview with Alinta Sara, 22 September 2020; Anionwu, ‘Health Education and Community Development for Sickle Cell Disorders in Brent’, 253.

57 Beverley Bryan, Stella Dadzie and Suzanne Scafe, *The Heart of the Race: Black Women’s Lives in Britain* (London, 1985), 108–9

58 Catherine (pseudonym), interview with author, 9 December 2018.

59 Chris Sirrs, ‘The Moment of Patient Safety: Iatrogenic Injury and Cultures of Healthcare in the NHS, c.1989-2000’, work-in-progress paper presented at the Centre for History of Medicine, University of Warwick (2021), 10.

60 This dynamic continues in the present—a 2015 patient survey found that over 50 per cent of SCD patients across the UK continue to have negative care experiences in emergency settings, reporting that emergency healthcare professionals had insufficient understanding of SCD, were unsympathetic, and did not ease the pain of SCD crisis swiftly enough. See Picker Institute, *Piloting a new Patient Reported Experience Measure for Sickle Cell Disease: A Report of the Findings* (Oxford, 2015), 19–22.

61 Alex Mold, ‘Repositioning the Patient: Patient Organisations, Consumerism, and Autonomy in Britain during the 1960s and 1970s’, *Bulletin of the History of Medicine*, 87 (2013), 226.

62 The National Archives (TNA), Kew, JA418/7N/Z/1, ‘Patient Perception Booklets’, 18 October 1993.

63 The UK’s first genetic screening programme, for the metabolic disorder phenylketonuria (PKU), had been rolled out in 1968. The 1970s onwards also saw the development of regional genetics centres which sought to integrate genetic services into the NHS. See William Leeming, ‘Ideas about Heredity, Genetics, and ‘Medical Genetics’ in Britain, 1900-1982’, *Studies in History and Philosophy of Biological and Biomedical Sciences*, 36 (2005), 538–58.

64 Roberta Bivins, ‘Coming ‘Home’ to (post)Colonial Medicine: Treating Tropical Bodies in Post-War Britain’, *Social History of Medicine*, 26 (2013), 9.

65 Royal Anthropological Institute, *Sickle Cell Anaemia in Nigeria*, prod. Ralph Hendrickse (London, 1964).

66 C. Brown, *Black and White Britain* (London, 1984), 1.

67 A. C. Stevenson and B. C. C. Davison, *Genetic Counselling* (London, 1976).

68 Home Office, ‘First Report from the Select Committee on Race Relations and Immigration, Session 1977-78, Vol II’ (London, 1978), 121.

69 Milica Brozovič, interview with author, 22 October 2018.

70 Stuart Hall et al., *Policing the Crisis: Mugging, the State, and Law and Order* (London and Basingstoke, 1978), 45.

71 Milica Brozovič, interview with author, 22 October 2018.

72 Catherine (pseudonym), interview with author, 9 December 2018.

73 Catherine (pseudonym), interview with author, 9 December 2018; Simon Dyson, ‘“Race”, Ethnicity and Haemoglobin Disorders’, *Social Science & Medicine*, 47 (1998), 123.

74 Anionwu, ‘Health Education and Community Development for Sickle Cell Disorders in Brent,’ 235–7.

75 D. I. K. Evans and V. M. Blair, ‘Neonatal Screening for Haemoglobinopathy: Results in 7691 Manchester Newborns’, *Archives of Disease in Childhood*, 51 (1976), 130.

76 Bailkin, *The Afterlife of Empire*, 175.

77 Tracey Reynolds, ‘Exploring the Absent/Present Dilemma: Black Fathers, Family Relationships, and Social Capital in Britain’, *The Annals of the American Academy of Political and Social Science*, (2009), 17–18. This also reflects wider attitudes towards unmarried mothers in this period, who Pat Thane and Tanya Evans have shown were automatically regarded by local authorities as ‘problem’ families, frequently allocated the most substandard housing, and targeted by benefit cuts during the 1980s. See Pat Thane and Tanya Evans, *Sinners? Scroungers? Saints? Unmarried Motherhood in Twentieth Century England* (Oxford, 2012), 172, 177.

78 D.I.K. Evans, ‘Letter: Screening for Sickle-Cell Disease’, *British Medical Journal*, 5937 (1974), 162.

79 Clare, *An OSCAR for My Troubles*, 95.

80 BCA 14/05/F ‘Sickle Cell Anaemia’, Spearhead, May 1976.

81 Stuart Hall, ‘From Scarman to Stephen Lawrence’, *History Workshop Journal*, 48 (1999), 194.

82 Usha Prashar, Elizabeth Anionwu and Milica Brozovič, *Sickle Cell Anaemia – Who Cares? A Survey of Screening and Counselling Services in England* (London, 1985): 46; Anionwu, ‘Health Education and Community Development for Sickle Cell Disorders in Brent,’ 100 and 202; and Milica Brozovič, interview with author, 22 October 2018.

83 Helen Valier and Roberta Bivins, ‘Organisation, Ethnicity and the British National Health Service’, in *Innovations in Health and Medicine: Diffusion and Resistance in the Twentieth Century*, ed. Jennifer Stanton (London and New York, 2002), 52–6.

84 Department of Health, *Report of a Working Party of the Standing Medical Advisory Committee on Sickle Cell, Thalassaemia and other Haemoglobinopathies* (London, 1993), 31.

85 British Society for Haematology, ‘Guidelines for Haemoglobinopathy Screening’, *Clinical and Laboratory Haematology*, 10 (1988), 88.

86 Christina Potrykus, ‘Sickle Cell: Call for Better Screening and Universal Services’, *Health Visitor*, 64 (December 1991), 404–5.

87 Otto Saumarez Smith, ‘The Inner City Crisis and the End of Urban Modernism in 1970s Britain’, *Twentieth Century British History*, 27 (2018), 578–98.

88 Parliamentary Home Affairs Committee, *Fifth Report: Summary of Recommendations* (London, 1981), xcv.

89 Jenny Bourne, ‘“May We Bring Harmony?” Thatcher’s Legacy on Race’, *Race & Class*, 55 (2013), 89.

90 Parliamentary Home Affairs Committee, *Race Relations and Immigration Sub-Committee, Session 1981-82: Minutes of Evidence* (London, 1982), 31.

91 Parliamentary Home Affairs Committee, *Race Relations and Immigration Sub-Committee*, 32–41.

92 Prashar et al, *Sickle Cell Anaemia*, 31.

93 This research proposal focused on the docklands area of Liverpool, and received £20,750 revenue funding in 1982. TNA AT81/278/2, ‘Proposal for funding: The health needs of ethnic minorities’.

94 Bourne, ‘‘May we bring Harmony’?’, 89. Rosalind Wild argues that the Urban Programme/ICP was an idea modelled on similar US initiatives, with the intention of neutralising Black radicalism with controlled state funding. Rosalind Wild, ‘Black was the Colour of our Fight’, unpublished PhD thesis (University of Sheffield, 2008), 173.

95 Ruth Schwarz Cowan, *Heredity and Hope: The Case for Genetic Screening* (Cambridge, Massachusetts, 2008), 237.

96 L. A. Reynolds and E. M. Tansey, *Wellcome Witness Seminar: History of Cervical Cancer and the Role of the Human Papillomavirus, 1960-2000* (London, 2009).

97 Bivins, ‘Immigration, Ethnicity and ‘Public’ Health Policy in Postcolonial Britain’, 128, 139.

98 A 1979 survey of SCD in Britain found 1,300 cases treated in 12 months, but the authors admitted that the number could be double that. L. R. Davis et al., ‘Survey of Sickle-Cell Disease in England and Wales’, *British Medical Journal*, 283 (1981), 1519–21.

99 Prashar et al., *Sickle Cell Anaemia*, 29.

100 A. Streetly, R. Latinovic, K. Hall and J. Henthorn, ‘Implementation of Universal Newborn Bloodspot Screening for Sickle Cell Disease and Other Clinically Significant Haemoglobinopathies in England: Screening Results for 2005-7’, *Journal of Clinical Pathology*, 62 (2009), 26–30.

101 BCA, RC/RF/17/02/C, ‘Note on inquest on Stephen Bogle (sickle cell patient) who died at Thames Magistrates Court on 12^th^ August 1986’, 20 January 1987.

102 Bryan et al., *The Heart of the Race*, 89.

103 Allan McNaught, *Health Action and Ethnic Minorities* (London, 1987), 8–10.

104 Sickle Cell Society Archive, London, ‘Sickle Cell News Review’ (May 1993).

## Notes

I am grateful to my supervisors, Margot Finn and Helga Satzinger. Thanks also go to Florence Sutcliffe-Braithwaite, Alex Mold, Rebecca Jennings, Caroline Bressey, Ayesha Nathoo, Peder Clark and Jack Saunders, who offered valuable comments on versions of this article. Many thanks to the people who gave their time to be interviewed as part of this work. I would like to thank the anonymous referees of TCBH whose generous engagement greatly improved this piece. This research was carried out with the support of the Wellcome Trust and the Royal Historical Society. Research for this article was conducted with the support of a Wellcome Doctoral Studentship, grant number 108601/Z/15/Z.

